# Suitability of anaerobic fungi culture supernatant or mixed ruminal fluid as novel silage additives

**DOI:** 10.1007/s00253-022-12157-w

**Published:** 2022-09-14

**Authors:** Thomas Hartinger, Katerina Fliegerová, Qendrim Zebeli

**Affiliations:** 1 Institute of Animal Nutrition and Functional Plant Compounds, Department for Farm Animals and Veterinary Public Health, University of Veterinary Medicine Vienna, Veterinärplatz 1, 1210 Vienna, Austria; 2 Laboratory of Anaerobic Microbiology, Institute of Animal Physiology and Genetics, Czech Academy of Sciences, 14220 Prague, Czech Republic

**Keywords:** Anaerobic fungi, Enzymes, Forage, Ruminant, Silage additive

## Abstract

**Abstract:**

This study investigated silage quality characteristics and ruminal fiber degradability of grass and straw ensiled with either anaerobic fungi (AF) supernatant with active fungal enzymes or mixed ruminal fluid as novel silage additives. Compared to control silages, AF supernatant improved the quality of grass and straw silages as evidenced by decreased pH, acetic acid concentration, and dry matter losses. Likewise, mixed ruminal fluid enhanced lactic acid fermentation, which further resulted in lower pH of the treated grass silage. The ruminal fiber degradability was determined using in situ incubations and, compared to controls, the cellulose degradability was higher for grass silage with AF supernatant, whereas ruminal degradability of straw silage was reduced by this treatment. In contrast, mixed ruminal fluid did not influence fiber degradability of silages in the rumen. Concluding, both novel additives improved silage quality, whereas only AF supernatant enhanced ruminal fiber degradability of grass silage and therefore may represent an approach for improving forage utilization by ruminants.

**Key points:**

*• Enzymes of anaerobic fungi supernatant improve quality of grass and straw silages.*

*• Mixed ruminal fluid enhances lactic acid fermentation when ensiling grass and straw.*

*• Enzymes of anaerobic fungi supernatant increase ruminal grass silage degradability.*

## Introduction

From physiological, ecological, and economical perspectives, silages constitute the main dietary component in cattle feeding, providing energy, nutrients, and structural fiber. An excellent silage quality with high ruminal degradability is of utmost importance to adequately meet the cattle’s nutritive demands for performance while decreasing the inclusion of concentrates in the diet. However, various abiotic and biotic factors may often cause poor forage quality that is characterized by fewer sugars but more recalcitrant fibrous structures, which eventually reduce the nutritive value and ruminal degradability of these forages (Lauer 2012; Borreani et al. 2018) and therefore jeopardize the adequate energy and nutrient exploitation by the animal. In this context, the future trend towards higher aridity in Europe (Forzieri et al. 2014) will increase the risk of drought-impaired and thus low-quality forage, which in turn emphasize the necessity for developing strategies that ensure the sufficient energy supply from silages of different substrate qualities.

Remarkably, the ensiling process itself has been demonstrated to slightly improve fiber degradability by deconstructing lignocellulosic complexes (Ambye-Jensen et al. 2013; Zhao et al. 2018). The use of novel silage additives based on enzymes originating from anaerobic fungi (AF) culture medium has been recently suggested as a way to more effectively pre-cleave fibrous structures in silages (Hartinger and Zebeli 2021). Anaerobic fungi are commensals of the rumen microbiome and efficiently degrade fiber using various enzymes as well as physical penetration via fungal hyphae (Hess et al. 2020). So far, only few studies investigated the use of viable fungal cultures during ensiling of rice straw and whole-crop corn. Thereby, treatment with viable fungal cultures resulted in enhanced lactic acid fermentation as well as higher ruminal degradability for both substrates (Lee et al. 2015; Wang et al. 2019). However, there is credible evidence that those improvements in AF-treated silages were not related to the viable AF cultures as they cannot survive in the silo (Lee et al. 2015), therefore rather suggesting no physical penetration of plant particles by fungal hyphae. More likely, the extracellularly fungal enzymes present in the culture medium (Haitjema et al. 2014. 2017) caused those beneficial effects observed (Hartinger and Zebeli 2021). Due to their wide pH range and affinity for recalcitrant fiber components (Hagen et al. 2020; Wang et al. 2019), AF enzymes may indeed pre-cleave fibrous compounds during ensiling and subsequently allow an enhanced degradability of silages in the rumen. Besides, activity of AF enzymes can release glucose from fiber breakdown (Wang et al. 2011), which in consequence could support lactic acid fermentation in the silo and thereby improve substrate conservation. Therefore, the first aim of our study was to investigate the effects of the addition of AF supernatant with fungal enzymes on the composition and in situ fiber degradability of grass or wheat straw silages, i.e., a common forage in cattle feeding and a by-product of grain production that is very rich in recalcitrant fiber. We hypothesized a stronger lactic acid fermentation in the silos as well as a higher in situ degradability due to activity of fungal enzymes when AF supernatant is included as a silage additive.

Since the isolation and cultivation of AF are complex and laborious (Dollhofer et al. 2015), a direct inoculation with ruminal fluid, which includes the rumen microbiome’s diverse enzymatic repertoire and consequently as well AF enzymes (Puniya et al. 2015), might constitute an analogous approach to tackle plant fiber structures already during ensiling. Up to now, ruminal fluid has not been explored as a silage additive, but in fact represents a reservoir of fibrolytics and their enzymatic tools, which may be beneficially used for ensiling. Therefore, a second aim of the study was to evaluate the suitability of mixed ruminal fluid as an inoculant for grass and straw silages. Compared to control silages, both a higher lactic acid fermentation and in situ degradability of silages treated with mixed ruminal fluid were expected.

## Materials and methods

### Preparation of anaerobic fungi supernatant as silage additive

For receiving the AF enzymes-based additive, i.e., the AF supernatant, an AF mixture isolated from the ovine rumen was anaerobically cultivated at 39 °C on M10 medium (Caldwell and Bryant 1966) enriched by 25% (v/v) rumen fluid with 4 g/l of xylan (Serva Electrophoresis GmbH, Heidelberg, Germany) as carbon source. The AF mixture was then incubated in 100-ml bottles for 4 days, mycelia were collected by centrifugation at 4000 × g for 20 min at 4 °C, and culture broth, i.e., AF supernatant, was further processed. The AF supernatant was tenfold concentrated by ultrafiltration (EMD Millipore 5121 Amicon Stirred Cell Model 8010, Cole-Parmer, Vernon Hills, IL, USA) under helium pressure of 300 kPa using membrane disc filters (B10K 76MM HIGH FLUX 10PK, Merck, Darmstadt, Germany) and subsequently lyophilized (Lyovac GT2, Leybold Heraeus, Cologne, Germany). The lyophilized powder was stored at − 20 °C until its application. The eligibility of AF supernatant was evaluated by determination of endo-1,4-β-xylanase (EC 3.2.1.8) and β-D-glucoside glucohydrolase (E.C.3.2.1.21) activity according to methods of Lever (1977) and Bidochka et al. (1993), respectively. The enzymatic activities of β-D-glucoside glucohydrolase and endo-1,4-β-xylanase were confirmed to be 261.8 nkat (170 µg of glucose/ml/h) and 1567.3 µkat (4,120 μg of xylose/ml/h), respectively.

For the strain-specific identification of present AF, DNA from the cultivated mycelium was isolated using DNeasy PowerSoil Kit (Qiagen, Hilden, Germany) and the whole ITS region was amplified using primer pair ITS1F (CTTGGTCATTTAGAGGAAGTAA; Gardes and Bruns 1993) and ITS4R (TCCTCCGCTTATTGATATGC; White et al. 1990), resulting in amplicons of approximately 740 bp length. The AF identification was based on clone library approach as described previously by Mura et al. (2019). Briefly, plasmid DNA was isolated from 30 randomly selected ITS clones of *Escherichia coli* using a GenElute™ HP Plasmid Miniprep Kit (Sigma-Aldrich, Burlington, MA, USA) and sent for Sanger sequencing using the M13F priming sites within the vector pCR4 (SEQme, Dobříš, Czech Republic). The sequences obtained from the cultivated AF mixture had highest similarity with three species of fungal genus *Neocallimastix*, i.e., *Neocallimastix* sp. NYR4 (GenBank accession number JQ782548, sequence identity 99.18%), *Neocallimastix* sp. WI3-B (GenBank accession number MK397979, sequence identity 97.57%), and *Neocallimastix* sp. NYF3 (GenBank accession number JQ782544, sequence identity 97.49%).

### Preparation of mixed ruminal fluid as silage additive

The mixed ruminal fluid was obtained from a rumen-cannulated dry Holstein cow fed grass silage and grass hay (70:30, dry matter (DM) basis) ad libitum and additionally 1 kg of concentrate mixture (i.e., ground corn, barley, and wheat in a ratio of 0.5:0.25:0.25) per day that was provided in two equal portions in the morning and afternoon. The cow was fed and kept according to the Austrian guidelines of animal welfare (BGBl. II Nr. 485/2004 idF BGBl. II Nr. 151/2017). Directly before ensiling, solid ruminal digesta was collected from the middle of the fiber mat and squeezed through three layers of gauze (Wilhelm Weisweiler GmbH & Co. KG, Münster, Germany) to obtain particle-associated mixed ruminal fluid that was directly applied to the respective silages.

### Preparation of straw and grass silages

For the experiment, silages were produced in 0.85-L glass jars (Weck GmbH u. Co. KG, Wehr, Germany) using either wilted grass [G] or wheat straw [S] as ensiling substrates. The chemical compositions of both substrates are presented in Table 1. The grass stand mainly consisting of perennial ryegrass (*Lolium perenne*) and meadow fescue (*Festuca pratensis*) was harvested as first cut at mid vegetative stage (inflorescence emergence) using a disc mower at the research dairy farm of the University of Veterinary Medicine Vienna (Pottenstein, Austria). Thereby, a rather fiber-rich grass stand was intentionally selected to better study the fiber-cleaving effects of the novel silage additives. All silages were prepared with approximately 35% DM concentration and a compaction density of 212.5 kg DM/m^3^ as recommended by the German Federal Working Group for Forage Preservation (Bundesarbeitskreis Futterkonservierung 2011). Thus, tap water was added to wheat straw before ensiling, whereas grass was wilted in the sun to adjust the DM concentration of plant material. Subsequently, the grass was chopped to 7 cm length before ensiling, while wheat straw was obtained from straw bales without mold or visible contamination and was already chopped to 7 cm particle length. Since straw is lacking water-soluble carbohydrates (WSC), which constitute the metabolizable substrate for lactic acid bacteria and consequently are pivotal for a sufficient acidification in the silo (McDonald et al. 1991), 18 g sucrose was added to each glass jar, i.e., constituting 10% of DM ensiled.

**Table 1:** Chemical composition of grass and wheat straw before ensiling

	Grass	Wheat straw
Dry matter (DM) concentration^1^, g/kg	422	931
Ash, g/kg DM	101	68.0
Crude protein, g/kg DM	182	39.7
Ether extract, g/kg DM	20.1	11.6
aNDFom^2^, g/kg DM	643	842
ADFom^3^, g/kg DM	286	473
ADL^4^, g/kg DM	53.2	119
WSC^5^, g/kg DM	167	66.6

^1^Determined after wilting of grass and before water addition to wheat straw

^2^Neutral detergent fiber assayed with a heat stable α-amylase and expressed exclusive of residual ash

^3^Acid detergent fiber expressed exclusive of residual ash

^4^Acid detergent lignin

^5^Water-soluble carbohydrates

Regarding the applied treatments, both grass and straw were ensiled with either (a) 18 mL of heat-inactivated fungal enzyme solution serving as a control (CON_AF), (b) 18 mL of freshly prepared anaerobic fungal enzyme solution (AF), (c) 18 mL of heat-inactivated mixed ruminal fluid serving as a control (CON_RF), or (d) 18 mL of freshly collected mixed ruminal fluid (RF). Therefore, the lyophilized powder, which contained the anaerobic fungal enzymes, was dissolved in distilled water and directly applied to the respective silages. The distilled water was added in such a quantity that the same volume of supernatant was again obtained as before freeze-drying of fungal culture supernatant. For the control treatments, both mixed ruminal fluid as well as dissolved AF supernatant were heat-inactivated by placing both into an oven at 103 °C for 4 h and subsequent cooling down to approximately 25 °C before being used as silage additives. The pH of the fresh and heat-inactivated fungal enzyme solution as well as the fresh and heat-inactivated mixed ruminal fluid was 8.84, 8.37, 6.89, and 6.74, respectively. Consequently, eight different silage treatments were produced, which are referred to as G_CON_AF, G_CON_RF, S_CON_AF, S_CON_RF, G_AF, S_AF, G_RF, and S_RF. Each silage treatment was prepared in triplicate and subsequently stored at 20 ± 1.3 °C for 90 days. All silos were weighed on day 1 and day 90 to calculate the DM loss. Additionally, fresh samples of grass and wheat straw were collected directly before ensiling and kept at − 20 °C until further analysis.

### Analysis of silage composition

Before analysis, samples of fresh substrates and silages were dried at 65 °C in a forced-air oven for 48 h and subsequently ground through a 1-mm screen in an ultra-centrifugal mill (ZM 200, Retsch, Haan, Germany). The nutrient analyses were conducted in accordance with guidelines of the Association of German Agricultural Analytic and Research Institutes (VDLUFA 2012). The DM concentration was determined by oven-drying the samples at 103 °C for at least 4 h (method 3.1) and, for silages only, corrected for drying losses of volatile compounds using the equation for grass silage of Weißbach and Kuhla (1995). The ash concentration was determined by combustion in a muffle furnace overnight at 580 °C (method 8.1); crude protein was analyzed using the Kjeldahl method (method 4.1.1) and ether extract using the Soxhlet extraction system (method 5.1.2). Regarding fiber fractions, proportions of neutral detergent fiber assayed with a heat stable α-amylase and expressed exclusive of residual ash (aNDFom), acid detergent fiber expressed exclusive of residual ash (ADFom), and acid detergent lignin (ADL) was determined in a Fibretherm FT12 (Gerhardt GmbH & Co. KG, Königswinter, Germany) according with methods 6.5.1, 6.5.2, and 6.5.3, respectively. The WSC concentration was analyzed in accordance with method 7.1.1.

### Analysis of silage fermentation pattern

Directly after silo opening, cold-water extracts of all silages were prepared by mixing 50 g of silage sample with 100 mL of distilled water and placing it for 16 h in the fridge at 4 °C. Subsequently, the complete content was filtered through three layers of gauze (Wilhelm Weisweiler GmbH & Co. KG, Münster, Germany) and pH was immediately determined potentiometrically (S40-K SevenMulti™ pH meter, Mettler Toledo, Vienna, Austria) in the liquid before being stored in aliquots at − 20 °C until further analyses.

The concentrations of volatile fatty acids acetate, propionate, butyrate, and ethanol were determined on a gas chromatography device (GC Model 8060 MS 172 DPFC, No.: 950713, Fisons, Rodano, Italy), equipped with a flame-ionization detector and a 30-m × 0.53-mm ID × 0.53-μm df capillary column (Trace TR Wax, Thermo Fisher Scientific, Vienna, Austria). The detailed protocol, including sample preparation, can be obtained from Hartinger et al. (2022). The ammonia concentration was analyzed using the Berthelot reaction (Hinds and Lowe 1980) and lactic acid concentration was determined by high-performance liquid chromatography (UltiMate 3000 HPLC system, Thermo Fisher Scientific, Vienna, Austria) according to Weiß and Kaiser (1995).

### Determination of in situ degradability

The effective ruminal degradability of DM, aNDFom, and ADFom of straw and grass silages as well as fresh straw and grass was determined applying the in situ nylon bag technique with a reduced number of incubation time points (Olaisen et al. 2003). Three runs were performed using the rumen-cannulated dry Holstein cow from which the mixed ruminal fluid has been obtained. Beginning 14 days prior to the first nylon bag incubation, the cow was continuously fed a basal diet of grass silage and corn silage ad libitum (65:35, DM basis), which was provided in the morning and afternoon. Likewise, the cow was additionally fed 1 kg of concentrate mixture per day (i.e., ground corn, barley, and wheat in a ratio of 0.5:0.25:0.25) that was also provided in two equal portions with the basal diet.

For the in situ incubations, each feedstuff, i.e., silages and fresh substrates, was dried at 60 °C for 48 h and ground through a 3-mm screen in an ultra-centrifugal mill (ZM 200, Retsch, Haan, Germany). Subsequently, 10 g DM was weighed into 10-cm × 20-cm polyester bags with a pore size of 50 ± 10 µm (R1020, ANKOM Technology, Macedon, NY, USA). Directly before being incubated in the rumen, duplicate samples of each feedstuff were placed in water (39 °C) for approximately 10 min with gentle movement of bags. Afterwards, all bags were inserted into the ventral sac of the rumen for 4 h, 12 h, 24 h, and 72 h. At the end of each incubation period, bags were removed and put in ice water to immediately stop microbial activity. Subsequently, all bags were washed on a cold rinse cycle in a washing machine for 30 min, dried at 60 °C for 48 h and analyzed for DM, aNDFom and ADFom as outlined before. Additionally, each run included “0 h bags” per feedstuff, which were directly removed after pre-soaking in water, i.e., not incubated into the rumen, and subsequently treated similar as described for the other bags.

The calculations of ruminal degradability of DM, aNDFom, and ADFom were performed using the equation of McDonald (1981):

$$\text{Ruminal degradability}=\mathrm{a}+\mathrm{b }\times (1-{e}^{-c \times \left(\mathrm{t}-\mathrm{L}\right)})$$

where *a* constitutes the fraction that disappears from the bag immediately, *b* constitutes the insoluble but potentially rumen-degradable fraction, *c* is the constant rate of disappearance of fraction *b*, *t* is the incubation period, and *L* represents the lag phase. These non-linear parameters, i.e., *a*, *b*, *c*, and *L*, were estimated using an iterative least squares procedure in SAS version 9.4 (SAS Institute Inc., Cary, NC, USA). The effective degradability of DM, aNDFom, and ADFom was estimated assuming a ruminal passage rate of 4%/h, as recommended for forages (Offner et al. 2003) using the equation:

$$\text{Effective ruminal degradability }= a + b \times c/(c + 0.04)$$

### Statistical analysis

Statistical analysis of silage composition and fermentation pattern variables was performed with the GLM procedure of SAS version 9.4 (SAS Institute Inc., Cary, NC, USA) using the following model:

$$Y= \mu + {p}_{i}+ {t}_{j}+(p \times {t)}_{ij}+ {e}_{ij}$$

where *µ* is the mean, *p*_*i*_ is the main effect of ensiled plant material, *t*_*j*_ is the main effect of treatment, (*p* × *t*)_*ij*_ is the two-way interaction between the main effects, and *e*_*ij*_ is the residual error. Differences between least square means were analyzed by Tukey–Kramer post hoc test. For the in situ degradability data, statistical analysis was performed with the MIXED procedure of SAS version 9.4 (SAS Institute Inc., Cary, NC, USA) using the following model:

$$Y=\mu+p_i+t_j+(p\times{t)}_{ij}+r_k+e_{ijk}$$

where *µ* is the mean, *p*_*i*_ is the fixed effect of ensiled plant material, *t*_*j*_ is the fixed effect of treatment, (*p* × *t*)_*ij*_ is the two-way interaction between the main effects, *r*_*k*_ is the random effect of run, and *e*_*ijk*_ is the residual error. Differences between least square means were again analyzed by Tukey–Kramer post hoc test. The significance level was set at *α* = 0.05, and a trend was declared at 0.05 < *P* < 0.10 for all analyses.

## Results

### Dry matter loss

The AF supernatant tendentially lowered the DM losses (*P* = 0.06) for grass and straw silages as evidenced by 2.20%, 1.34%, 1.97%, and 1.82% DM losses for G_CON_AF, G_AF, S_CON_AF, and S_AF, respectively (standard error of the mean = 0.30). The substrate (*P* = 0.59) and its interaction with AF supernatant treatment (*P* = 0.16) had both no effect on DM losses.

Regarding the grass and straw silages treated with mixed ruminal fluid, DM losses were lower in grass silages than in straw silages (*P* < 0.01), i.e., 1.49%, 1.68%, 2.22%, and 2.68% for G_CON_RF, G_RF S_CON_RF, and S_RF, respectively (standard error of the mean = 0.21). Treating silages with mixed ruminal fluid (*P* = 0.13) as well as its interaction with substrate (*P* = 0.46) had no influence on DM losses.

### Nutritional composition and fermentation pattern of silages

The nutritional composition and fermentation pattern of grass and straw silages treated with AF supernatant are presented in Table 2. The DM concentration of grass silages was approximately 6 percentage points higher than in straw silages (*P* < 0.01). The AF supernatant reduced the ash concentration in straw silages, whereas no differences were observed in grass silages (*P* < 0.01). Likewise, ash concentration was higher in G_AF than S_AF (*P* < 0.01) and generally higher in grass silages than in straw silages (*P* < 0.01). Regarding crude protein, the concentrations were higher in G_AF than G_CON_AF, but not differing between straw silages (*P* < 0.01), which had a generally lower crude protein concentration than grass silages (*P* = 0.01). An interaction of AF supernatant treatment and substrate was present for ether extract with higher values for grass silages compared to straw silages (*P* = 0.02). Besides, ether extract concentration was higher in grass silages compared to straw silages, i.e., 26.5 vs. 11.2 g/kg DM, as evidenced by a substrate effect (*P* < 0.01). The straw silages had higher concentrations of aNDFom and ADFom than grass silages (*P* < 0.01), but besides this substrate-specific difference, no effects were found in terms of fiber fractions (each *P* > 0.05). Treating silages with AF supernatant reduced the pH by around 0.3 units (*P* = 0.04). Besides, pH in grass silages was on a higher level than in straw silages (*P* < 0.01), i.e., 4.72 vs. 3.87, respectively. The straw silages treated with AF supernatant had less acetic acid compared to its control, whereas acetic acid concentrations in grass silages were similar (*P* < 0.01). Additionally, a substrate effect was observed (*P* < 0.01), revealing that acetic acid was lower abundant in grass silages than in straw silages. Similarly, propionic acid concentration was also lower in grass silages than in straw silages (*P* = 0.03). Regarding ethanol, a substrate effect was observed with higher concentrations in straw than in grass silages (*P* = 0.04), whereas AF supernatant or its interaction with substrate had no impact. The ammonia proportion was generally increased by the addition of AF supernatant (*P* < 0.01) with higher values in S_AF than S_CON_AF (*P* = 0.03). Besides, concentrations of ammonia were lower in grass silages than in straw silages (*P* < 0.01). No effects of AF supernatant treatment, substrate or their interaction were found for concentrations of ADL, WSC, lactic acid, and butyric acid (each *P* > 0.05).

**Table 2:** Effect of anaerobic fungi supernatant on nutritional composition and fermentation pattern of silages

	Grass silage		Straw silage		SEM	*P*-values		
	CON_AF^1^	AF^2^	CON_AF	AF		Treatment	Substrate	Interaction
DM^3^ concentration, g/kg	429	392	335	338	1.65	0.10	< 0.01	0.07
Ash, g/kg DM	97.0	111^A^	89.0^a^	59.3^bB^	2.90	0.03	< 0.01	< 0.01
Crude protein, g/kg DM	183^b^	192^a^	39.3	41.2	1.01	< 0.01	< 0.01	0.01
Ether extract, g/kg DM	24.5^A^	28.5^A^	12.2^B^	10.2^B^	0.89	0.30	< 0.01	0.02
aNDFom^4^, g/kg DM	613	599	793	810	14.2	0.95	< 0.01	0.32
ADFom^5^, g/kg DM	311	308	494	504	5.61	0.54	< 0.01	0.31
ADL^6^, g/kg DM	42.8	61.3	58.6	64.5	6.77	0.11	0.19	0.37
WSC^7^, g/kg DM	33.0	38.2	59.3	38.4	18.7	0.69	0.51	0.51
pH	4.89	4.54	4.01	3.73	0.13	0.04	< 0.01	0.80
Lactic acid, g/kg DM	33.5	54.0	26.5	24.9	10.4	0.40	0.13	0.33
Acetic acid, g/kg DM	7.63	9.36	25.8^a^	15.5^b^	1.00	< 0.01	< 0.01	< 0.01
Butyric acid, g/kg DM	0.00	0.00	0.13	0.83	0.20	0.16	0.06	0.16
Propionic acid, g/kg DM	0.00	0.00	5.70	1.90	1.34	0.20	0.03	0.20
Ethanol, g/kg DM	1.82	2.49	5.23	4.20	0.97	0.86	0.04	0.42
Ammonia, g/kg N	44.6	52.3	76.6^b^	97.8^a^	2.45	< 0.01	< 0.01	0.03

In each row, superscript capitalized letters indicate difference (*P* < 0.05) between substrates within each treatment, i.e., CON_AF and AF, and superscript lowercase letters indicate difference (*P* < 0.05) between treatments within each substrate, i.e., straw and grass

^1^Silage prepared with 18 mL of heat-inactivated anaerobic fungi supernatant

^2^Silage prepared with 18 mL of freshly prepared anaerobic fungi supernatant

^3^Dry matter

^4^Neutral detergent fiber assayed with a heat stable α-amylase and expressed exclusive of residual ash

^5^Acid detergent fiber expressed exclusive of residual ash

^6^Acid detergent lignin

^7^Water-soluble carbohydrates

Both the nutritional composition and fermentation pattern of grass and straw silages treated with mixed ruminal fluid are presented in Table 3. Regarding the nutritional composition, mixed ruminal fluid reduced the ADL concentration compared to control silages, i.e., 64.4 vs. 118 g/kg DM (*P* < 0.01), and tended to increase the ADFom concentration in silages (*P* = 0.08). Moreover, the substrate affected several nutritional composition variables with higher concentrations of DM, ash, crude protein, and ether extract in grass silages compared to straw silages (each *P* < 0.01), whereas all fiber fractions were higher in straw silages than in grass silages (each *P* < 0.01). Regarding the silage fermentation pattern, mixed ruminal fluid lowered the pH in grass silages, whereas no differences were present within straw silages, but pH of S_CON_RF was lower than of G_CON_RF (*P* = 0.01). Silage pH was also affected by main effects of mixed ruminal fluid treatment and substrate with higher pH in control silages (*P* = 0.04) and grass silages (*P* < 0.01), respectively. Similar to pH results, the acetic acid concentration in mixed ruminal fluid-treated grass silages was higher than in control grass silages but not differing between straw silages (*P* = 0.05). Contrastingly, the ammonia concentration was higher in S_RF compared to S_CON_RF with similar values in grass silages (*P* = 0.04). Besides, main effects of mixed ruminal fluid treatment and substrate revealed generally lower ammonia proportions in control silages (*P* = 0.01) and grass silages (*P* < 0.01), respectively. The addition of mixed ruminal fluid increased the lactic acid concentration in both grass and straw silages (*P* = 0.01). Moreover, grass silages tended to have more lactic acid compared to straw silages (*P* = 0.07). The propionic acid concentration was higher in mixed ruminal fluid-treated straw silages than control straw silages (*P* = 0.03); plus within mixed ruminal fluid-treated silages, straw silage contained more propionic acid than grass silage (*P* = 0.03). Besides, main effects of mixed ruminal fluid treatment and substrate were observed with higher propionic acid values for mixed ruminal fluid treatment than control (*P* < 0.01) and straw silages than grass silages (*P* < 0.01), respectively. The ethanol concentration was affected by the interaction of mixed ruminal fluid and substrate (*P* = 0.03) with higher values in control straw silage than in control grass silage. Besides, a main substrate effect was observed for this variable and straw silages contained more ethanol than grass silages (*P* = 0.01). Effects of mixed ruminal fluid treatment, substrate, or their interaction were not found for either WSC or butyric acid concentration (each *P* > 0.05).

**Table 3:** Effect of mixed ruminal fluid on nutritional composition, and fermentation pattern of silages

	Grass silage		Straw silage		SEM	*P*-﻿values		
	CON_RF^1^	RF^2^	CON_RF	RF		Treatment	Substrate	Interaction
DM^3^ concentration, g/kg	409	386	342	348	0.80	0.31	< 0.01	0.13
Ash, g/kg DM	105	111	68.1	63.6	2.84	0.78	< 0.01	0.11
Crude protein, g/kg DM	187	190	36.1	45.4	3.56	0.14	< 0.01	0.35
Ether extract, g/kg DM	26.7	28.7	13.0	10.8	1.38	0.94	< 0.01	0.18
aNDFom^4^, g/kg DM	553	576	832	842	8.78	0.10	< 0.01	0.50
ADFom^5^, g/kg DM	315	326	500	512	5.70	0.08	< 0.01	0.94
ADL^6^, g/kg DM	93.1	47.3	142	81.4	6.70	< 0.01	< 0.01	0.30
WSC^7^, g/kg DM	20.6	28.3	52.4	19.6	18.1	0.49	0.52	0.29
pH	4.60^aB^	4.29^b^	4.19^A^	4.25	0.05	0.04	< 0.01	0.01
Lactic acid, g/kg DM	22.7	84.87	8.85	46.0	11.7	0.01	0.07	0.30
Acetic acid, g/kg DM	16.2^b^	29.6^a^	21.7	20.0	2.91	0.09	0.48	0.05
Butyric acid, g/kg DM	0.00	0.00	8.69	4.93	0.41	0.65	0.11	0.59
Propionic acid, g/kg DM	0.17	0.97^B^	1.27^b^	4.13^aA^	0.35	< 0.01	< 0.01	0.03
Ethanol, g/kg DM	2.55^B^	4.60	8.44^A^	5.83	0.76	0.71	0.01	0.03
Ammonia, g/kg N	47.5	53.0	77.7^b^	104.8^a^	4.59	0.01	< 0.01	0.04

In each row, superscript capitalized letters indicate difference (*P* < 0.05) between substrates within each treatment, i.e., CON_AF and AF, and superscript lowercase letters indicate difference (*P* < 0.05) between treatments within each substrate, i.e., straw and grass

^1^Silage prepared with 18 mL of heat-inactivated mixed ruminal fluid

^2^Silage prepared with 18 mL of freshly collected mixed ruminal fluid

^3^Dry matter

^4^Neutral detergent fiber assayed with a heat stable α-amylase and expressed exclusive of residual ash

^5^Acid detergent fiber expressed exclusive of residual ash

^6^Acid detergent lignin

^7^Water-soluble carbohydrates

### In situ degradability

**Fig. 1:**
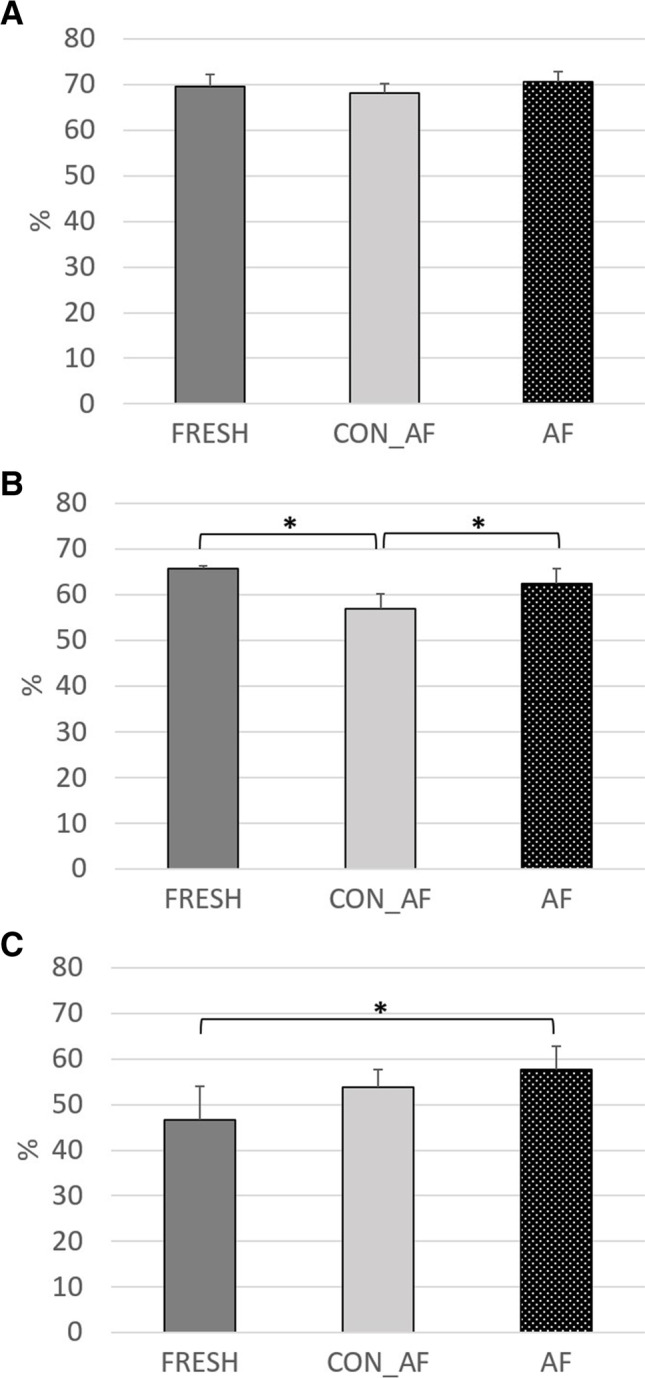
Effective ruminal degradability (4%/h passage rate) of dry matter (**A**), neutral detergent fiber (**B**), and acid detergent fiber (**C**) of original fresh grass (FRESH) or grass silages prepared with heat-inactivated (CON_AF) or freshly prepared anaerobic fungi supernatant (AF). Asterisk = *P* < 0.05 in Tukey–Kramer post hoc test

**Fig. 2:**
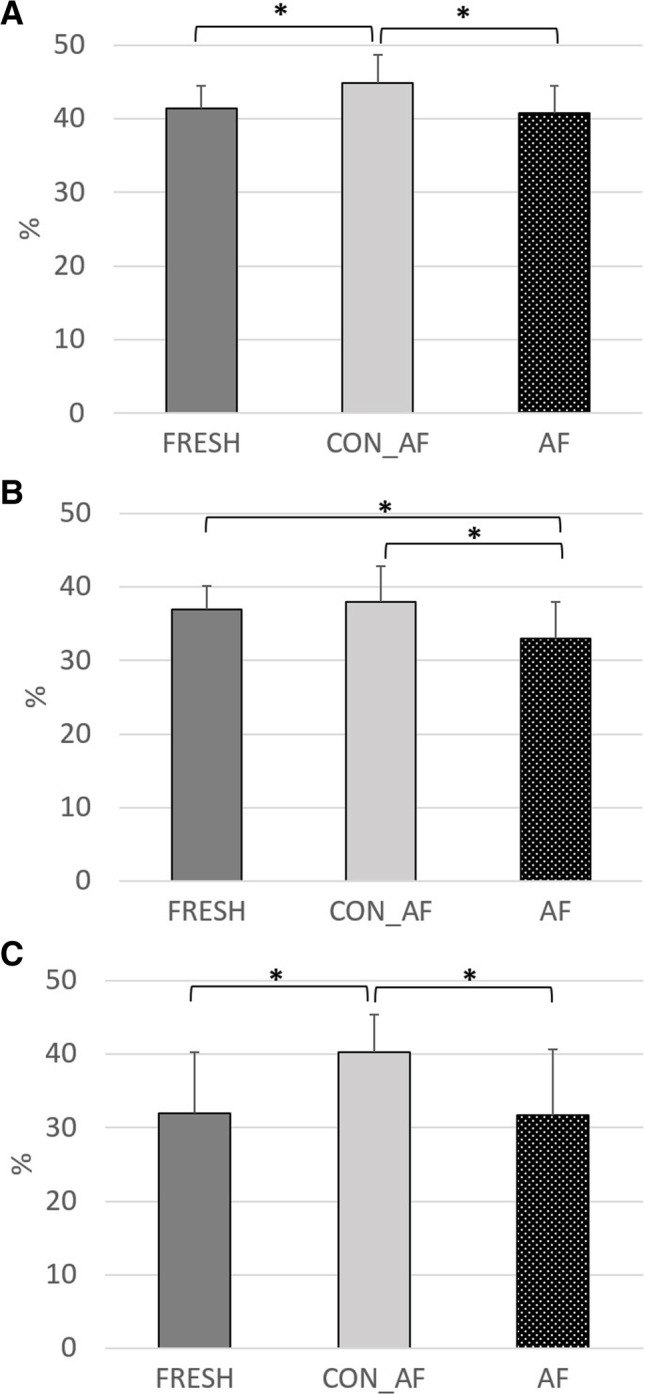
Effective ruminal degradability (4%/h passage rate) of dry matter (**A**), neutral detergent fiber (**B**), and acid detergent fiber (**C**) of original fresh straw (FRESH) or straw silages prepared with heat-inactivated (CON_AF) or freshly prepared anaerobic fungi supernatant (AF). Asterisk = *P* < 0.05 in Tukey–Kramer post hoc test

The results of effective ruminal degradability regarding the use of AF supernatant as a silage additive are presented in Figs. 1 and 2; the respective data for constants a, b, and c and lag phase can be obtained from Table 4. The DM degradability was not different between G_CON_AF, G_AF, and fresh grass (Fig. 1A), whereas an interaction of substrate and AF supernatant treatment for DM degradability was observed (*P* < 0.01) with higher values for S_CON_AF than S_AF and fresh straw (Fig. 2A). Besides, also substrate affected DM degradability with higher values for grass than for straw (*P* < 0.01). No main effect on DM degradability was observed for AF supernatant (*P* = 0.55). An interaction of substrate and AF supernatant treatment was also present for the aNDFom degradability (*P* < 0.01) with lower values for S_AF than S_CON_AF and fresh straw (Fig. 2B), whereas for grass, aNDFom degradability was lower for G_CON_AF than G_AF and fresh grass (Fig. 1B). Moreover, main effects of substrate (*P* < 0.01) and AF supernatant (*P* < 0.01) were significant for ruminal aNDFom degradability. Regarding the degradability of ADFom, interaction of substrate and AF supernatant was significant (*P* = 0.01) with higher ADFom degradability for G_AF than for fresh grass with G_CON_AF being intermediate (Fig. 1C), whereas ADFom degradability of S_CON_AF was higher when compared to S_AF and fresh straw (Fig. 2C). As found for aNDFom degradability, main effects of substrate (*P* < 0.01) and AF supernatant (*P* = 0.02) were also significant for ADFom degradability.

**Table 4:** Constants of ruminal degradation for fresh grass (G_FRESH), grass silage treated with heat-inactivated anaerobic fungi supernatant (G_CON_AF), grass silage treated with freshly prepared anaerobic fungi supernatant (G_AF), fresh straw (S_FRESH), straw silage treated with heat-inactivated anaerobic fungi supernatant (S_CON_AF), and straw silage treated with freshly prepared anaerobic fungi supernatant (S_AF)

	G_FRESH	G_CON_AF	G_AF	S_FRESH	S_CON_AF	S_AF
Dry matter						
* a*^1^	43.64	40.31	42.38	17.52	21.50	20.42
* b*^2^	43.46	48.63	48.10	57.67	58.34	54.45
* c*^3^, %/h	0.06	0.05	0.06	0.03	0.03	0.03
Lag time	2.90	2.59	2.14	4.90	4.70	4.40
Neutral detergent fiber assayed with a heat stable α-amylase and expressed exclusive of residual ash						
* a*	37.59	23.28	30.32	10.59	11.64	9.79
* b*	51.16	63.96	58.20	71.16	67.59	69.60
* c*, %/h	0.05	0.04	0.05	0.03	0.03	0.02
Lag time	3.87	3.82	4.43	4.90	4.90	4.80
Acid detergent fiber expressed exclusive of residual ash						
* a*	16.47	20.15	23.12	7.50	16.83	13.00
* b*	81.41	66.00	62.74	62.16	61.16	52.75
* c*, %/h	0.03	0.04	0.05	0.02	0.03	0.02
Lag time	5.26	4.30	3.60	4.50	4.90	4.40

^1^Fraction that disappears from the bag immediately

^2^Insoluble but potentially rumen-degradable fraction

^3^Constant degradation rate of fraction *b*

**Fig. 3:**
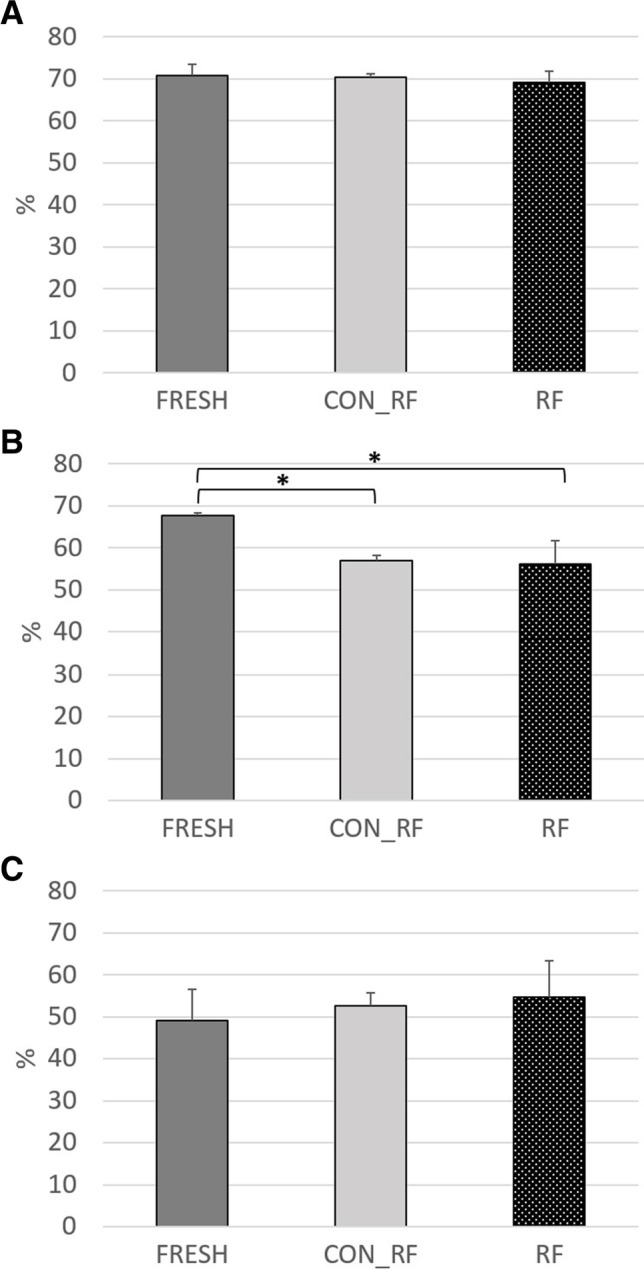
Effective ruminal degradability (4%/h passage rate) of dry matter (**A**), neutral detergent fiber (**B**), and acid detergent fiber (**C**) of fresh grass (FRESH) or grass silages prepared with heat-inactivated (CON_RF) or freshly prepared mixed ruminal fluid (RF). Asterisk = *P* < 0.05 in Tukey–Kramer post hoc test

**Fig. 4:**
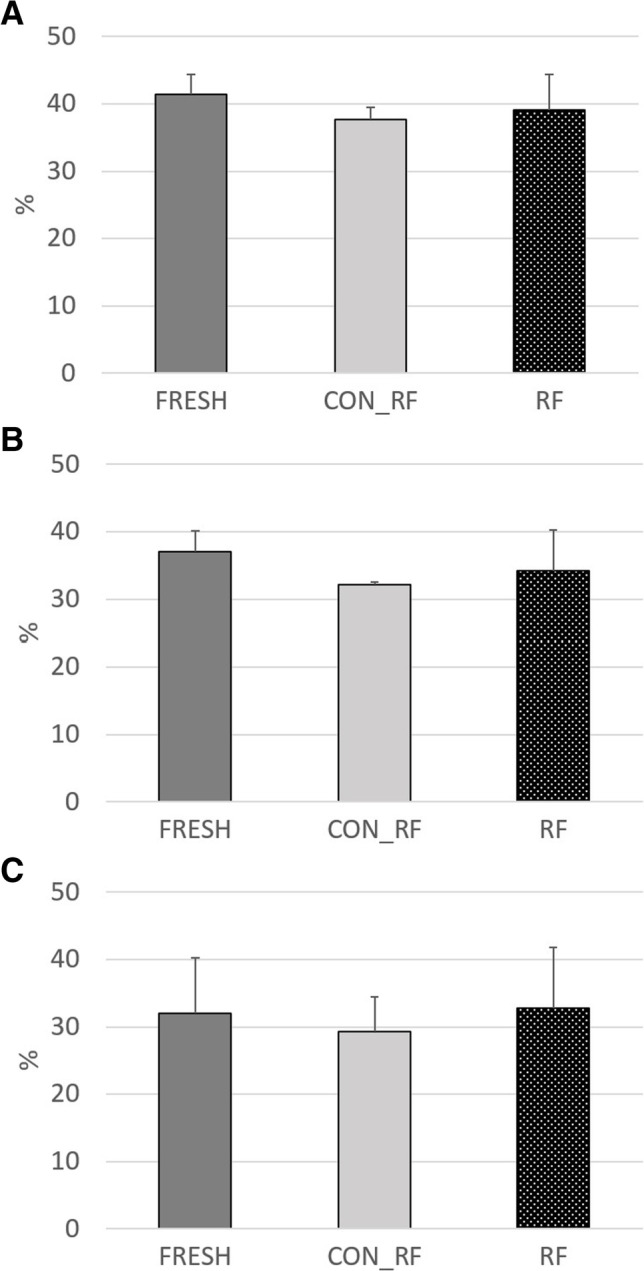
Effective ruminal degradability (4%/h passage rate) of dry matter (**A**), neutral detergent fiber (**B**) and acid detergent fiber (**C**) of fresh straw (FRESH) or straw silages prepared with heat-inactivated (CON_RF) or freshly prepared mixed ruminal fluid (RF). Asterisk = *P* < 0.05 in Tukey–Kramer post hoc test

The results of effective ruminal degradability regarding the use of mixed ruminal fluid as a silage inoculant are shown in Figs. 3 and 4; the respective data for constants a, b, and c and lag phase can be obtained from Table 5. Hereby, the effective ruminal DM degradability was influenced by substrate (*P* < 0.01) with generally higher values for grass than for straw. The mixed ruminal fluid treatment (*P* = 0.28) as well as the interaction of substrate and mixed ruminal fluid (*P* = 0.45) showed no differences in DM degradability between incubated feedstuffs (Figs. 3A and 4A). For aNDFom degradability, a trend for the interaction of substrate and mixed ruminal fluid was found (*P* = 0.08) with higher values for fresh grass compared to G_CON_RF and G_RF (Fig. 3B), but similar values for fresh straw, S_CON_RF, and S_RF (Fig. 4B). Besides, substrate (*P* < 0.01) and mixed ruminal fluid (*P* < 0.01) affected the aNDFom degradability. As found for DM degradability, ADFom degradability was influenced by substrate (*P* < 0.01) with generally higher values for grass than for straw, whereas neither the mixed ruminal fluid treatment (*P* = 0.41) nor its interaction with substrate (*P* = 0.45) impacted the ADF degradability of incubated feedstuffs (Figs. 3C and 4C).

**Table 5:** Constants of ruminal degradation for fresh grass (G_FRESH), grass silage treated with heat-inactivated mixed ruminal fluid (G_CON_RF), grass silage treated with freshly collected mixed ruminal fluid (G_RF), fresh straw (S_FRESH), straw silage treated with heat-inactivated mixed ruminal fluid (S_CON_RF), and straw silage treated with freshly collected mixed ruminal fluid (S_RF)

	G_FRESH	G_CON_RF	G_RF	S_FRESH	S_CON_RF	S_RF
Dry matter						
* a*^1^	43.64	42.52	41.00	17.52	15.76	17.04
* b*^2^	43.46	45.70	49.47	57.67	55.91	50.59
* c*^3^, %/h	0.06	0.06	0.05	0.03	0.02	0.04
Lag time	2.90	3.48	3.50	4.90	4.90	3.50
Neutral detergent fiber assayed with a heat stable α-amylase and expressed exclusive of residual ash						
* a*	37.59	22.55	19.50	10.59	6.71	10.96
* b*	51.16	66.61	69.28	71.16	76.03	62.14
* c*, %/h	0.05	0.04	0.05	0.03	0.02	0.03
Lag time	3.87	4.90	4.00	4.90	4.90	4.50
Acid detergent fiber expressed exclusive of residual ash						
* a*	16.47	21.68	20.08	7.50	6.94	11.17
* b*	81.41	68.66	65.38	62.16	59.91	48.97
* c*, %/h	0.03	0.04	0.05	0.02	0.01	0.03
Lag time	5.26	4.90	3.48	4.50	4.90	3.80

^1^Fraction that disappears from the bag immediately

^2^Insoluble but potentially rumen-degradable fraction

^3^Constant degradation rate of fraction *b*

## Discussion

The present study investigated the impact of AF supernatant with active fungal enzymes on composition and fermentation quality of grass and straw silages, i.e., a typical and a recalcitrant fiber-rich forage, and determined their ruminal DM and fiber degradability. We hypothesized a stronger lactic acid fermentation during ensiling as well as a higher ruminal fiber degradability due to enhanced cleavage of structural carbohydrates by AF supernatant in the silo.

Regarding silage quality, the addition of AF supernatant lowered the pH in all silages compared to respective controls, and in case of grass silage, the treatment reduced the silage pH to 4.54 and thus below the DM-dependent threshold for stable conservation (Muck 1988). Although the lactic acid level was not significantly affected, the numerical increase of 20.5 g/kg DM in AF supernatant-treated grass silages seemed to — at least partly — explain this observed pH decline. Likewise, the AF supernatant decreased the acetic acid concentration in straw silages without affecting the lactic acid concentration, indicating a shift towards a homolactic-dominated fermentation (Borreani et al. 2018). Consequently, these beneficial influences on silage fermentation characteristics revealed an improved silage quality and forage preservation with AF supernatant, which was also reflected by the trend for reduced DM losses in both grass and straw silages. Therefore, our hypothesis of a stronger lactic acid fermentation may not be confirmed, but the overall improved silage quality in response to AF supernatant indeed suggest a beneficial effect of AF supernatant in silages.

Interestingly, the addition of AF supernatant resulted in an increased ammonia concentration in straw silages, suggesting higher protein degradation in the silo. It is conceivable that the AF supernatant as well included proteases as the applied AF supernatant was obtained from species of *Neocallimastix*, a genus whose members partly show proteolytic activity, as well (Hartinger et al. 2018). Such AF-induced proteolysis is believed to be associated with the degradation of structural proteins to sufficiently decompose fibrous plant structures, and it may further modify the activities of other fungus-derived CAZymes (Wallace and Joblin 1985). Therefore, a certain proportion of proteolytic activity in AF cultures may be inevitable, but in terms of fiber degradation supportive. However, it has to be noted that ammonia proportion still amounted for less than 10% of total nitrogen in AF supernatant-treated straw silages, which is deemed as a sufficient true protein conservation (Kung et al. 2018). Similarly, no difference in ammonia concentrations between control and AF supernatant-treated silages was observed for grass, meaning our positive assessment of AF supernatant for silage quality holds true.

The proximate nutrients of the silages were mainly affected by the ensiled substrate, i.e., grass or straw, with expectedly higher concentrations of ash, crude protein and ether extract, but lower concentrations of fiber fractions in grass than straw, as it has also been found in the fresh substrates. The overall aNDFom concentrations were decreased after ensiling, which may be related to the acidic hydrolysis of hemicelluloses in the silo (Dewar et al. 1963). The treatment with AF supernatant, however, showed no influence on the fiber fraction concentrations, meaning that a potential fiber tackling effect of AF enzymes was not reflected in silage composition. This is in contrast to prior studies inoculating rice straw or whole-plant corn with various viable AF species before ensiling, which observed less NDF and ADF concentrations compared to controls (Lee et al. 2015; Wang et al. 2019).

The ruminal degradability of fiber fractions, however, was indeed affected by this treatment. Accordingly, our most important finding here was the improved fiber degradability of grass silages when ensiled with AF supernatant: The aNDFom degradability of AF supernatant-treated grass silages was higher than of control grass silages and similar to fresh grass, while ruminal ADFom degradability, i.e., ruminal lignocellulose degradation, was even higher for AF supernatant-treated grass silages when compared to fresh grass. Thus, our hypothesis was confirmed and it can be assumed that the enzymes present in the AF supernatant pre-cleaved lignocellulosic complexes in the silos, thus allowing a higher fiber degradability, especially cellulose, and eventually higher energy exploitation from grass silage in the rumen. As outlined in our companion paper (Hartinger and Zebeli 2021), AF comprise a large enzymatic spectrum, and apart from β-glucosidase and endoxylanase, which have been considered in our study, more enzymes or cellulosomes have likely been active in the applied AF supernatant. For instance, using transcriptomics and proteomics, Wang et al. (2011) identified several novel fiber-degrading enzymes in *Neocallimastix patriciarum* W5. Thus, similar omics-based approaches can help to characterize the AF enzymes and consequently also to understand the modes of action in the silo.

In case of straw silages, the beneficial effects of AF supernatant seen for silage quality could not be transferred to ruminal degradation. Surprisingly, the control straw silages, i.e., prepared with heat-inactivated AF supernatant, had higher DM, aNDFom, and ADFom degradabilities than straw ensiled with AF supernatant and further investigations are needed to explore the mechanisms behind. Moreover, ruminal degradability of DM and ADFom was also higher for control straw silages when compared to fresh straw, thus still supporting the previously described improvements in fiber degradability by the process of ensiling (Ambye-Jensen et al. 2013; Zhao et al. 2018). Our findings on the effect of AF supernatant in straw silages are not in line with Lee et al. (2015), who observed higher ruminal fiber degradability when ensiling rice straw with viable AF cultures. In contrast, wheat straw was ensiled in the present study, and apart from a difference between the application of viable AF cultures or their supernatant, a straw type-specific influence is possible and may be taken into account.

Focusing only on the application of AF supernatant as a silage additive, our results clearly showed a substrate-specific influence on the efficacy of AF supernatant in silages, i.e., grass vs. straw. The AF supernatant improved the quality of both grass and straw silages, but beneficial effects on ruminal fiber degradability were only present for grass silages. Thus, our hypothesis of a higher ruminal degradability of forages ensiled with AF supernatant was confirmed for grass silages only. Studies investigating the impact of AF supernatant in other common forages, such as corn and alfalfa, can provide further information on substrate-specificities and therefore better define the potential areas of application. Likewise, refinements in the culturing and preparation of AF or their supernatant are required to further increase the efficacy of this novel silage additive. Hereby, co-culturing of AF and methanogens may constitute an option to additionally increase the fibrolytic enzyme yield as suggested by the observed upregulated transcription of CAZymes in such co-cultures (Swift et al. 2019). Likewise, as previous data indicate differences in AF species regarding their effectiveness as silage inoculants (Wang et al. 2019; Lee et al. 2015), research on applying supernatants from further AF species or genera seems rationale — although it may be noted that the present AF supernatant was obtained from a culture of three *Neocallimastix* species, a genus that is considered to express highest enzyme activities among AF cultures (Dagar et al. 2018), particularly after sequential sub-culturing (Ekinci et al, 2006). In regard to esterase activity, being mainly responsible for the breakup of lignocellulosic complexes, a collection of AF supernatant after 1 or 2 days of cultivation may enhance its fiber cleaving impact as a silage inoculant, especially in straw silages, since esterase activities were observed to be highest during early fungal growth, whereas cellulase and xylanase peak at a later time point of incubation (Dagar et al. 2018).

As a second part, our study further assessed the use of mixed ruminal fluid as a silage additive in grass and straw silages, which may inoculate the silo with fibrolytic microbes and their enzymes that in consequence tackle fiber components during silo storage. Provided a similar efficacy as when using AF supernatant for ensiling, the approach of directly applying mixed ruminal fluid would mean a reduction in complexity, time, and labor compared to the production of AF supernatant (Dollhofer et al. 2015). Consequently, we analyzed the same parameters as for AF supernatant-treated silages and expected an improved silage fermentation and subsequent ruminal fiber degradability in response to the microorganisms and enzymes deriving from the mixed ruminal fluid.

The higher lactic acid concentrations in silages treated with mixed ruminal fluid indeed demonstrated an enhanced lactic acid fermentation. Likewise, addition of mixed ruminal fluid reduced the pH to 4.29 in grass silages and therefore indicated a stable forage conservation (Muck 1988). Compared to control, the acetic acid concentration was doubled in grass silages with mixed ruminal fluid, i.e., 16.2 vs. 29.6 g/kg DM, which suggested a higher activity of heterolactic lactobacilli or ruminal fluid-derived acetate producers. However, as shown by the lack of differences in DM losses, an acetic acid level of around 30 g/kg DM may not cause energy losses, but can actually be interpreted as beneficially in terms of aerobic stability due to yeast inhibitory effects (Danner et al. 2003). Likewise, the overall low ethanol levels indicate a general reduced presence and suppression of epiphytic yeasts in all silages (Kung et al. 2018), therefore supporting the assumed sufficient aerobic stability. The slightly higher ammonia concentrations observed in treated straw silages may have been caused by proteolytic microbes brought into the silo via mixed ruminal fluid (Hartinger et al. 2018; Puniya et al. 2015). Thus, the effects of mixed ruminal fluid on ammonia formation in straw silages were the same as those of AF supernatant and appear to have a similar rationale. In parts, the higher ammonia may have also originated from the mixed ruminal fluid itself (Puniya et al. 2015) and vaporized during heat inactivation, thus co-explaining the lower ammonia level in control straw silages. Still, the ammonia concentration of 10.5% of total nitrogen in mixed ruminal fluid-treated straw silages appears uncritical in regards to true protein conservation (Kung et al. 2018) and, consequently, our hypothesis of an improved silage fermentation with the addition of mixed ruminal fluid was confirmed.

Worth of notice is the apparently high lignin reduction with mixed ruminal fluid inoculation in both grass and straw silages, which has not been observed for the treatment of silages with AF supernatant. Since the rumen microbiota is incapable or only minimally able to degrade lignin (Susmel and Stefanon 1993), this observation was indeed very surprising and lacks a direct explanation. It might be conjectured that the mixed ruminal fluid-induced ADL degradation led to a higher availability of fermentable sugars and therefore would explain the increased lactic acid concentrations but absence of an effect of mixed ruminal fluid on WSC concentration — but this seems actually highly speculative. In consequence, ruminal fiber degradability should be increased if lignin was truly degraded (Zabel and Morrell 2020).

However, a beneficial effect of such a lignin reduction was not reflected in ruminal degradability. In fact, the DM and ADFom degradability was not influenced by mixed ruminal fluid in grass and straw silages and, for aNDFom, ruminal degradability was even lower after ensiling grass with fresh or heat-inactivated mixed ruminal fluid when compared to fresh grass. Thus, our hypothesis of an improved fiber degradation due to a pre-cleaving effect by ruminal fluid-derived microbes during ensiling could not be confirmed. However, as the DM degradability remained similar between fresh grass and grass silages, degradability of other nutrients must have compensated the reduction in aNDFom degradability, which may be investigated in future studies. Consequently, inoculation of grass and straw silages with mixed ruminal fluid may be suitable for improving silage quality, but for a fiber-cleaving activity in the silo, the targeted application of AF supernatant cannot be replaced by directly using mixed ruminal fluid. However, as demanded for AF supernatant, further research may help to optimize this approach, as well. For instance, a concentration of microorganisms and their enzymes via ultrafiltration of mixed ruminal fluid, like it has been performed during processing of the AF supernatant, could increase the fiber cleaving potential during ensiling. Since the present straw silages were prepared of pure wheat straw, applying mixed ruminal fluid as a silage inoculant in mixed silages of straw and a second substrate, e.g., grass or WSC-rich by-products, may be worth of further investigation.

In conclusion, this is the first study to investigate AF supernatant and mixed ruminal fluid as novel silage additives for ensiling grass and wheat straw and we provide strong evidence that both candidates improve silage quality. The treatment with AF supernatant additionally enhanced ruminal fiber degradability of grass silage, which should be associated with a fiber-cleaving fungal enzyme activity in the silo. Therefore, the application of AF supernatant in silages represents a promising strategy to support forage utilization by ruminants and should be pursued. Differences in ruminal degradability between wheat straw and grass silages suggest substrate-specific effects that need consideration in future research.

## Data Availability

All data generated or analyzed during this study are included in this published article.
